# *Agrobacterium* sp. ZX09 β-Glucan Attenuates Enterotoxigenic *Escherichia coli*-Induced Disruption of Intestinal Epithelium in Weaned Pigs

**DOI:** 10.3390/ijms231810290

**Published:** 2022-09-07

**Authors:** Yuankang Zhou, Yuheng Luo, Bing Yu, Ping Zheng, Jie Yu, Zhiqing Huang, Xiangbing Mao, Junqiu Luo, Hui Yan, Jun He

**Affiliations:** 1 Animal Nutrition Research Institute, Sichuan Agricultural University, Chengdu 611130, China; 2 Key Laboratory of Animal Disease-Resistance Nutrition, Chengdu 625014, China

**Keywords:** β-glucan, intestinal epithelium, enterotoxigenic *Escherichia coli*, weaned pigs

## Abstract

To explore the protective effect of dietary β-glucan (BGL) supplementation on intestinal epithelium exposure to enterotoxigenic *Escherichia coli* (ETEC), thirty-two weaned pigs were assigned to four groups. Pigs were fed with a basal diet or basal diet containing 500 mg/kg BGL, and were orally infused with ETEC or culture medium. Results showed BGL supplementation had no influence on growth performance in weaned pigs. However, BGL supplementation increased the absorption of D-xylose, and significantly decreased the serum concentrations of D-lactate and diamine oxidase (DAO) in the ETEC-challenged pigs (*p* < 0.05). Interestingly, BGL significantly increased the abundance of the zonula occludens-1-(ZO-1) in the jejunal epithelium upon ETEC challenge (*p* < 0.05). BGL supplementation also increased the number of S-phase cells and the number of sIgA-positive cells, but significantly decreased the number of total apoptotic cells in the jejunal epithelium upon ETEC challenge (*p* < 0.05). Moreover, BGL significantly increased the duodenal catalase (CAT) activity and the ileal total superoxide dismutase (T-SOD) activity in the ETEC-challenged pigs (*p* < 0.05). Importantly, BGL significantly decreased the expression levels of critical inflammation related proteins such as the tumor necrosis factor-α (TNF-α), interlukin-6 (*IL-6*), myeloid differentiation factor 88 (*MyD88*), and nuclear factor-κB (*NF-κB*) in the jejunal and ileal mucosa upon ETEC challenge (*p* < 0.05). BGL also elevated the propanoic acid content and the abundance of *Lactobacillus* and *Bacillus* in the colon upon ETEC challenge (*p* < 0.05). These results suggested BGL could alleviate the ETEC-induced intestinal epithelium injury, which may be associated with suppressed inflammation and improved intestinal immunity and antioxidant capacity, as well as the improved intestinal macrobiotic.

## 1. Introduction

The intestinal epithelium is the first line of defense for mammalian animals against external antigens and pathogenic microorganisms [1]. In pig production, changes in the diet form and removal of maternal antibody protection at weaning increases the susceptibility of post-weaning pigs to various pathogenic factors, which may lead to inflammation and injury of the intestinal epithelium [2,3]. Enterotoxigenic *Escherichia coli* (ETEC) is one of the major bacterial causes of post-weaning diarrhea, and accounts for more than 40% of the economic loss in pig production. ETEC colonizes the gut via fimbriae or fimbriae-related adhesins and secretes enterotoxins, which increases the levels of intracellular cAMP or cGMP in host epithelial cells and ultimately results in watery diarrhea [4]. Moreover, ETEC not only impairs the intestinal epithelial tight junction and increases the permeability of intestinal epithelium, but also induces overproduction of reactive oxygen species or apoptosis of the intestinal epithelial cells, which lead to injury of the intestinal barrier functions [5,6,7,8]. Various antibiotics have long been utilized to prevent the ETEC-induced diarrhea and intestinal inflammation. However, the development of drug resistance or residues has limited their use in animal production, and alternatives to the antibiotics have attracted considerable research interest worldwide [9].

β-glucans (BGL) are composed of glucose molecules linked together by a (1–3), (1,4) or (1,6) linear β-glycosidic chain, and different types of glucans may vary in length and branching structures [10]. BGL can activate adaptive and non-adaptive immune responses; however, the immune activity of BGL is associated with its structural complexity [11]. As a pathogen-associated molecular pattern (PAMP), BGL can be recognized by various membrane receptors (such as Dectine-1, CR3 and scavenger receptors), and activate a variety of signaling pathways to exert different functions [12,13]. For instance, BGL was found to inhibit the release of *IL-1β* upon *NLRP3* inflammasome activation [14], and BGL can also promote the maturation of tumor-educated dendritic cells (TEDCs), which subsequently enhances antitumor immune responses and inhibits tumor progression [15]. Moreover, BGL can act as a substrate for microbial fermentation and selectively stimulate the growth of some beneficial bacteria, such as the *Bifidobacterium* and *Lactobacillus* in the intestine [16]. These attributes should make the BGL an attractive candidate for substitution for antibiotics to prevent various intestinal inflammation or diseases.

The aim of this study was to explore the protective effect of dietary BGL supplementation on the intestinal epithelium in weaned pig exposure to ETEC. We found that BGL was capable of attenuating the ETEC-induced intestinal epithelium injury, which was associated with suppressed inflammation and improved intestinal immunity and antioxidant capacity, as well as the improved intestinal microbiota.

## 2. Results

### 2.1. Effect of BGL on Growth Performance in Weaned Pigs upon ETEC Challenge

There were no differences in ADG, ADFI, F:G ratio among the four treatments during the experiment period (*p* > 0.05) (Table 1).

### 2.2. Effect of BGL on Intestinal Permeability and Distribution of ZO-1 Protein in Weaned Pigs upon ETEC Challenge

**Figure 1:**
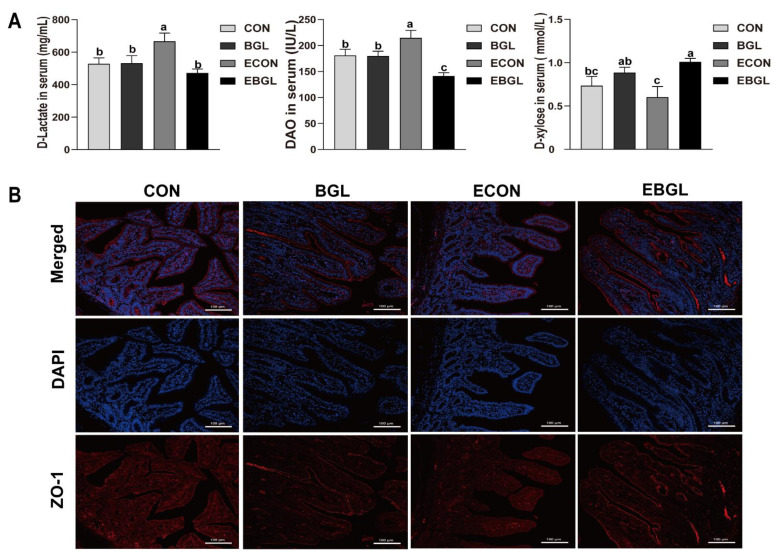
Effect of BGL on intestinal permeability (**A**) and distribution of ZO-1 protein (**B**) in weaned pigs upon ETEC challenge Zonula occludens 1, ZO-1; 4’-6-diamidino-2-phenylindole, DAPI. ZO-1 protein (red), DAPI stain (blue), and merged ZO-1 protein and DAPI are shown. a, b, c, values within a row with different superscript letters are significantly different (*p* < 0.05). CON, pigs were fed with a basal diet; BGL, pigs were fed with a BGL-containing diet (500 mg/kg); ECON, pigs were fed with a basal diet and challenged by ETEC; EBGL, pigs were fed with a BGL-containing diet (500 mg/kg) and challenged by ETEC.

ETEC challenge improved the serum D-lactic and DAO (*p* < 0.05). However, BGL supplementation decreased them in the serum, and BGL significantly improved the serum concentration of D-xylose upon ETEC challenge (*p* < 0.05) (Figure 1A). In addition, ZO-1 was highly expressed and localized on the apical intercellular region of the jejune epithelium after BGL supplementation (Figure 1B).

### 2.3. Effect of BGL on Cell Cycle and Apoptosis in the Intestinal Epithelial Cells

**Figure 2:**
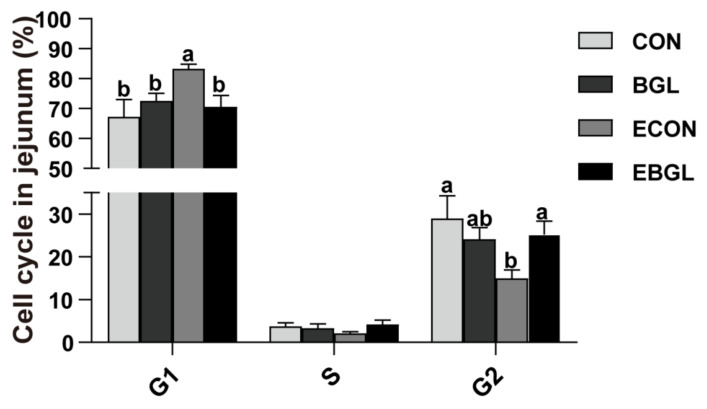
Effects of BGL supplementation on jejunal cell cycle (%) in weaned piglets upon ETEC challenge. a, b, values within a row with different superscript letters are significantly different (*p* < 0.05). CON, pigs were fed with a basal diet; BGL, pigs were fed with a BGL-containing diet (500 mg/kg); ECON, pigs were fed with a basal diet and challenged by ETEC; EBGL, pigs were fed with a BGL-containing diet and challenged by ETEC.

**Figure 3:**
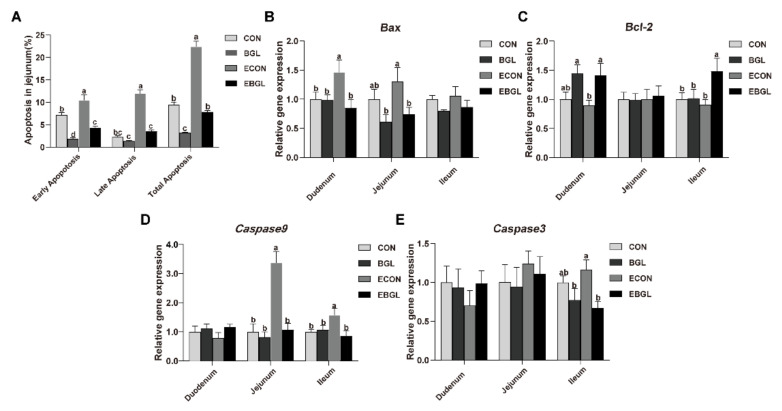
Effects of BGL on the percentage of apoptotic cells (**A**) and the relative expressions of related genes (**B**–**E**) in the small intestine of weaned pigs upon ETEC challenge. a, b, c, values within a row with different superscript letters are significantly different (*p* < 0.05). CON, pigs were fed with a basal diet; BGL, pigs were fed with a BGL-containing diet (500 mg/kg); ECON, pigs were fed with a basal diet and challenged by ETEC; EBGL, pigs were fed with a BGL-containing diet and challenged by ETEC.

BGL supplementation decreased the number of G_1_-phase cells, but significantly increased the number of S-phase cells in the upon ETEC challenge (*p* < 0.05) (Figure 2). ETEC challenge increased the jejunal epithelium ratio of total apoptotic cells (Figure 3). BGL supplementation significantly decreased the ratio of total apoptotic cells both in the ETEC-challenged and non-challenged pigs (*p* < 0.05). ETEC challenge significantly increased the jejunal expression level of *caspase 9* and the duodenal expression level of *Bax* (*p* < 0.05). However, BGL supplementation significantly decreased their expression levels upon ETEC challenge (*p* < 0.05). Moreover, BGL supplementation also decreased the ileal expression levels of *caspase 9* and *caspase 3*, but increased the expression level of *Bcl-2* upon ETEC challenge (*p* < 0.05).

### 2.4. Effect of BGL on sIgA Secretion in the Intestinal Mucosa

**Figure 4:**
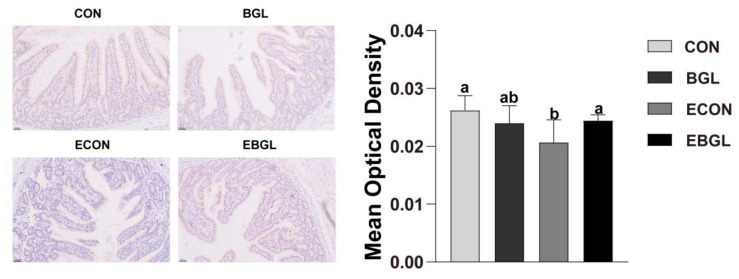
Effects of BGL on the sIgA content in the jejunum of weaned pigs upon ETEC challenge. (immunohistochemistry: ×100). Secretory immunoglobulin A, sIgA. a, b, values within a row with different superscript letters are significantly different (*p* < 0.05). CON, pigs were fed with a basal diet; BGL, pigs were fed with a BGL-containing diet (500 mg/kg); ECON, pigs were fed with a basal diet and challenged by ETEC; EBGL, pigs were fed with a BGL-containing diet and challenged by ETEC.

ETEC challenge decreased the jejunal epithelium number of sIgA positive cells (*p* < 0.05). However, BGL supplementation significantly increased it upon ETEC challenge (Figure 4).

### 2.5. Effect of BGL on Antioxidant Capacity of Intestinal Mucosa

BGL supplementation significantly decreased the concentration of MDA upon ETEC challenge (*p* < 0.05). BGL supplementation also increased the activity of CAT in the duodenal mucosa. Compared with CON group, the ileal activity of T-AOC of BGL group was higher. BGL supplementation also increased the activity of T-SOD and GSH-PX in the ileal mucosa upon ETEC challenge (*p* < 0.05) (Table 2).

### 2.6. Effect of BGL on Critical Genes Related to Intestinal Mucosal Inflammation and Intestinal Barrier Functions in Weaned Pigs upon ETEC Challenge

**Figure 5:**
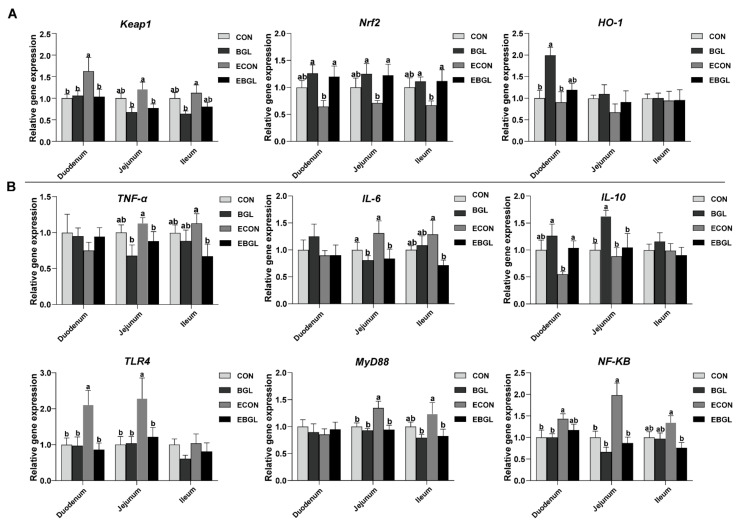
Effect of BGL on critical genes related to intestinal antioxidant capacity (**A**) and inflammation (**B**) in weaned pigs upon ETEC challenge. a, b, values within a row with different superscript letters are significantly different (*p* < 0.05). CON, pigs were fed with a basal diet; BGL, pigs were fed with a BGL-containing diet (500 mg/kg); ECON, pigs were fed with a basal diet and challenged by ETEC; EBGL, pigs were fed with a BGL-containing diet and challenged by ETEC. TNF-α, tumor necrosis factor-α; *IL-6*, interlukin-6; *IL-10*, interlukin-10; *MyD88*, myeloid differentiation factor 88; *NF-κB*, nuclear factor-κB; TLR4, Toll like receptor 4; *Nrf2*, nuclear factor erythroid 2-related factor 2; *HO-1*, Heme oxygenase-1; *Keap1*, Kelch-like ECH-associated protein 1.

BGL supplementation significantly increased the expression level of *Nrf2* in the ETEC-challenged pigs (*p* < 0.05). BGL supplementation decreased the jejunal and duodenal expression levels of *Keap1* (*p* < 0.05), the expression levels of *TNF-α* and *IL-6* in the jejunum and ileum upon ETEC challenge also decreased (*p* < 0.05). Moreover, BGL supplementation also increased the expression level of *IL-10* in the duodenum (*p* < 0.05). Importantly, BGL supplementation significantly decreased the expression levels of *TLR4, NF-κB*, and *MyD88* in the jejunum upon ETEC challenge (*p* < 0.05) (Figure 5).

### 2.7. Effect of BGL on Intestinal Microbial Populations and Metabolites in Weaned Pigs upon ETEC Challenge

ETEC challenge increased the abundance of *Escherichia coli* in the colon (*p* < 0.05). BGL supplementation significantly increased the abundance of *Lactobacillus* and *Bacillus* in the upon ETEC challenge (*p* < 0.05). Moreover, BGL supplementation also elevated the concentration of propanoic acid in the ETEC-challenged pigs (*p* < 0.05) (Table 3).

## 3. Discussion

In this study, BGL is extracted from *Agrobacterium* sp. *ZX09*, which has a large molecular structure. Some studies confirmed that dietary BGL supplementation can improved ADG and ADFI during the experiment [17,18]; however, no difference in growth performance was observed in our study. This may be different from the molecular dose and the content of BGL. Like other prebiotics, BGL can be resistant to enzymatic hydrolysis in the foregut, before entering the hindgut mostly intact [19], and BGL selectively stimulate the growth of beneficial gut bacteria to influence the host health [20]. In this study, BGL supplementation significantly increased the abundances of *Lactobacillus* and *Bacillus*, two classical beneficial microorganisms in the maintenance of intestinal immunity and epithelium functions [21,22,23]. Moreover, BGL increased the concentration of propanoic acid in the colon digesta upon ETEC challenge. Butyrate and propionate can suppress human monocyte derived DC activation by inhibiting the LPS-induced expression of the costimulatory molecule CD40 and secretion of IL-6 and IL-12p40 [24].

The small intestine is the first line of defense against various antigens that can elicit specific responses [25]. The intestinal epithelial cells directly protect the host against pathogenic microorganisms through tight junction structures [26]. Apical junction complex (AJC) composed of the tight junction (TJ) and adherens junction (AJ), is connected to the actomyosin cytoskeleton via zonula occludens (ZO) proteins and catenins, which can seal the space of the paracellular pathway [27]. The apical brush border structure of intestinal epithelium-specific ZO-1 knockout (KO) mice is disrupted, showing irregular microvillus length and diameter [28]. The intestinal barrier integrity was impaired upon ETEC challenge [29], and the serum activity of DAO and the concentration of D-lactic increased following intestinal mucosal injury or epithelial cell shedding [30]. In our study, their concentrations and the activity increased after ETEC challenge; however, BGL supplementation not only decreased their concentration and activity, but also enhanced the localization of ZO-1 and the concentration and D-xylose in the ETEC-challenged pigs. These results are consistent with previous studies on mice [31,32]. The improved integrity of the intestinal epithelium may be associated with two mechanisms. The first is that oligosaccharides including the BGL may prevent the adhesion of ETEC on the surface of intestinal epithelium, which significantly attenuates the ETEC-induced inflammation and injury in the intestine [33]. Moreover, BGL can be recognized by various receptors (such as Dectin-1, complement receptor 3, scavenger receptors CD5 and lactosylceramide), which subsequently induces a series of immune responses to eliminate pathogens. For instance, BGL can bind to dectin-1 to enhance phagocytosis of neutrophils and macrophages, or cooperate with TLRs to stimulate cytokine secretion which subsequently induces death of pathogenic bacteria [13,34]. These results suggested a protective effect of the BGL on the intestinal barrier in pigs upon ETEC challenge.

The cell cycle is usually divided into four phases (G1, S, G2, and M phase), and cell cycle checkpoints can repair problems in a timely manner to ensure normal cell growth. For instance, G_1_ arrest can prevent cells from initiating DNA replication. G2 arrest can prevent cells from entering the M phase in mammals [35]. Many bacterial pathogens (e.g., *Escherichia coli*, *Salmonella* Typhi) can produce the cytolethal distending toxin (CDT) or cycle inhibiting factor (Cif). The functional toxin protein cdtB of CDT can exhibit DNase I-like activity, resulting in DNA double-strand breaks, and the Cif also causes cellular G1/S and G2/M arrest without activating the DNA damage responses [36,37,38]. In this study, ETEC challenge induced G1 arrest in jejunum. However, BGL supplementation promoted cells into S phase to prevent G1 arrest.

Apoptosis is mainly initiated through the mitochondrial pathway and death receptor pathway, in which the protein family of caspases are an important participant, as they can act as both the initiator (e.g., *caspase 8* and *caspase 9*) and the final executor (such as *caspase3*, *caspase6* and *caspase7*) [39]. Upon pathogen infection or oxidative stress, the mitochondrial membrane permeability transition pore (MPTP) is opened, and the cytoplasmic cytC binds to Apaf-1 to form apoptotic bodies, which subsequently forms an apoptotic complex via binding to *caspase-9*, resulting in transmission of signals to downstream effector *(caspase3*) and initiation of apoptosis [40]. The balance of anti-apoptotic (e.g., *Bcl-2*, *Bcl-w*) and pro-apoptotic proteins (e.g., *Bax*, *Bak*) in the protein family of *Bcl-2* determines whether cells undergo apoptosis [41]. During late apoptosis, cells that are not phagocytosed by scavenger cells (e.g., macrophages) in a timely fashion are likely to undergo secondary necrosis or pyroptosis [42]. The activation of apoptotic *caspase3* induces secondary necrosis through cleavage of GSDMD and DFNA5, and the secondary necrosis is manifested by cell swelling and cytoplasmic membrane damage [43]. The massively released cellular contents can further stimulate the immune response as danger-associated molecular patterns (DAMPs) [43,44]. In this study, ETEC challenge elevated the apoptosis of the intestinal epithelium cells (IEC), which is consistent with a previous study on piglets [45]. Importantly, BGL supplementation significantly decreased the apoptosis rate, and decreased the expression levels of *caspase 9* and *BAX* in the jejunal epithelium. Lipopolysaccharide (LPS) is the main component of the outer membrane of *E. coli*, and its terminally abundant variable O-antigens are important for *E. coli* colonization and virulence [46]. Secretory immunoglobulin (sIgA) is the main immunoglobulin in the gut, and forms a microclimate with mucus and paneth cell products (such as defensins, lysozyme) in the intestinal lumen, and is responsible for preventing adhesion of bacteria to the epithelium [47]. Moreover, sIgA can neutralize bacterial toxins because of its high affinity for glycans (such as O antigens, polysaccharide capsules, and teichoic acid), which is distributed on the surface of gut bacteria [48,49]. Fernandez et al. (2003) found that sIgA could prevent LPS-induced translocation of *NF-κB* and subsequently down-regulated the expressions of proinflammatory factors (e.g., *TNF-α* and *MIP-2*) in IEC [50]. In this study, BGL significantly improved the abundance of sIgA in jejunum upon ETEC challenge, indicating an improved intestinal immunity in the ETEC-challenged pigs.

ETEC infection leads to an imbalance of oxidative and anti-oxidative systems, as featured by an increase in the levels of reactive oxygen species (ROS) [8]. Unscavenged ROS can disrupt protein conformation and produce lipid peroxides, resulting in damage of the structure and function in cells and tissues [51]. MDA is one of the end products of polyunsaturated fatty acid (PUFA) peroxidation, and is widely used as a biomarker of oxidative stress [52]. SOD and catalase (CAT) are two critical antioxidative enzymes in the body [53]. A previous study indicated that BGL has antioxidant properties, as it can provide hydrogen or electrons to eliminate free radicals [54]. In this study, BGL supplementation not only reduced MDA content in the small intestine, but also increased the activities of duodenal CAT and ileal T-SOD. THis result is consistent with a previous study on RAW264.7 cells [55]. Both results suggest that BGL can enhance intestinal antioxidant capacity by enhancing antioxidant enzyme activity.

Synthesis of antioxidant enzymes is tightly controlled by the nuclear factor erythroid 2-related factor 2 (*Nrf2*). Upon conformation changes of *Keap1* (inducers react with the cysteine residues of *Keap1*), the *Nrf2* dissociates from *Keap1* and translocates into the nucleus, where *NRF2* binds to antioxidant response element (ARE), which finally activates the expression of downstream antioxidant genes [56]. Heme oxygenase-1 (*HO-1*), the downstream product of *Nrf2*, can decompose heme into CO, free iron, and biliverdin [57]. In addition, *Keap1-Nrf2* signaling pathway is associated with anti-inflammatory effects. For instance, the *Nrf2* can inhibit transcriptional initiation of pro-inflammatory factors (*IL-6* and *IL-1β*) in mouse and human macrophages upon LPS-challenge, which is not dependent on reducing the production of ROS [58]. A previous study indicated that BGL can be partially internalized by microfolds of small intestinal M cells and released by immune organs in the form of a smaller fragment, leading to a series of mucosal immune responses [59]. TLRs are one of the important pattern recognition receptors (PRRs) for detecting invading pathogens in the gut. Importantly, ETEC is recognized by TLR4 and the activated *TLR4* activates *NF-κB* signaling pathway through the adaptor protein *Myd88* [60]. In this study, BGL supplementation significantly elevated the expression level of *Nrf2* in the intestinal mucosa upon ETEC challenge, which is consistent with the increases in the activities of major antioxidant enzymes. Moreover, BGL supplementation significantly decreased the expression levels of of *TLR4, NF-κB*, and *MyD88* in the jejunum. Both of these results indicated an anti-inflammatory effect of BGL on pigs challenged by ETEC.

## 4. Materials and Methods

### 4.1. Animal Diets and Experimental Design

All experimental protocols used in the animal experiment were approved by the Institutional Animal Care and Use Committee of Sichuan Agricultural University (No.20181105). ETEC (O149:K91, K88ac) was bought from the China veterinary culture collection center with a CVCC no. 225 (Beijing, China). *Agrobacterium* sp. ZX09 (Salecan^®^) used in this study was isolated from a soil sample from the ocean coast, β-glucan (the content ≥ 60%) was provided by SYNLGHT BIO Co., Ltd. *Agrobacterium* sp. ZX09 was cultured in HTM medium (1‰ potassium dihydrogen phosphate, 3‰ sodium nitrate, 0.2‰ magnesium sulfate, 0.07‰ calcium chloride, 0.0125‰ ferrous chloride, 20‰ sucrose, and 0.9% agar) at 28 °C for 48 h. The culture medium was precipitated by adding 95% ethanol, and the polysaccharides were collected by centrifugation at 6000× *g* for 15 min and dried at 60 °C. to the dried β-glucan was pulverized to a fine powder using a high-speed blender, and stored at room temperature [61].

Thirty-two Duroc x Landrace x Yorkshire piglets (boars) weaned at 21 days (6.82 ± 0.16 kg), were assigned into a 2 (BGL) × 2 (ETEC) factorial arrangement of four treatments composed of CON (basal diet), BGL (500 mg/kg β-glucan containing diet) and ECON (basal diet and challenged by ETEC), EBGL (500 mg/kg β-glucan and challenged by ETEC). The experiment lasted for 28 d; on 25–28 d, the ETEC challenge groups were orally dosed with 100 mL Luria Bertani (LB) medium containing 1 × 10^10^ CFU/mL ETEC every day; other groups were dosed with LB media of equal volume. During the experiment, pigs received the same parental nutrition and management. The corn–soybean basal diet (Table S1) was formulated according to National Research Council 2012 (NRC2012) [62]. Pigs were solitary in an about 1.5 × 0.7 m^2^ metabolism cage and temperature was controlled to between 27 and 30 °C, relative humidity 65% ± 5%.

### 4.2. Sample Collection

After 12 h fasting, blood samples were obtained in the morning on day 29 by precaval vein puncture, and the serum was centrifuged at 3500× *g* for 15 min and then packaged. Following blood collection, intestinal samples were collected after pigs were sacrificed with sodium pentobarbital (200 mg/kg BW). Duodenum, jejunum, and ileum segments of about 2–4 cm were taken and fixed in phosphate buffered saline (PBS) for flow cytometry or immunofluorescence and immunohistochemical analysis in 4% paraformaldehyde solution. Moreover, the intestinal mucosa was collected in a low temperature environment. Colon digesta samples were collected into sterile tubes. Mucosa and digesta samples were quick-frozen in liquid N_2_ and stored at −80 °C for long-term storage.

### 4.3. Growth Performance Evaluation

Piglets were weighed on d 1 and 29 after 12 h fasting, and the number of diarrhea pigs per day was recorded to assess piglet health status. Production performance-related indicators, such as average daily gain (ADG), average daily feed intake (ADFI), and the ratio of feed intake to weight gain (F:G) were calculated by recording feed intake and the weight change of pigs during the experiment period. Fresh excreta were ranked using the following scale: 0 = solid; 1 = semi-solid; 2 = semi-liquid; and 3 = liquid. The occurrence of diarrhea was defined as the maintenance of fecal scores of 2 or 3 for two consecutive days [63]. Diarrhea rate (%) = number of pigs with diarrhea within a treatment/(number of pigs × total observational days) × 100 [64].

### 4.4. Serum Parameter Measurement

The level of D-lactic acid and the activity of DAO were measured using porcine Enzyme-Linked Immunosorbent Assay (ELISA) kits (Shanghai Meimian Biotechnology Co., Ltd., Shanghai, China). The level of D-xylose was measured by assay kits (Nanjing Jiancheng Institute of Bioengineering, Nanjing, China).

### 4.5. Intestinal Antioxidant Parameters

Duodenal, jejunal and ileal mucosa were made into 10% homogenate, and Malondialdehyde (MDA), total antioxidant capacity (T-AOC), Glutathione peroxidase (GSH-Px), total superoxide dismutase (T-SOD), and catalase (CAT) were measured by corresponding assay kits (Nanjing Jiancheng Institute of Bioengineering, Nanjing, China).

### 4.6. Flow Cytometry Assays

#### 4.6.1. Cell Cycle Measurement

After preparation of jejunal epithelial cell suspension, a total of 1 mL cell suspension was transferred to a flow tube and centrifuged (250× *g*), and the supernatant discarded. The cells were permeabilized with 2 mL of 70% ethanol at 4 °C for 30 min, and the cells were washed with PBS twice. Then, 500 μL propidium iodide (PI)/Rnase staining Buffer was added and incubated at 4 °C for 30 min in the dark. Finally, after washing with PBS, 400 μL of PBS was added to the cell, and the cell cycle distributions were assayed by CytoFLEX flow cytometry (Beckman Coulter, Inc., Brea, CA, USA) within 45 min and analyzed using ModFit LT 5.0 software (Verity Software House, Topsham, ME, USA).

#### 4.6.2. Apoptosis Measurement

After centrifugation of the jejunal single-cell suspension, cells were suspended in 1× binding buffer. The resuspension was sequentially added with Annexin V-FITC fluorescent dye and PI for staining in the dark. Finally, the cells were resuspended in PBS and apoptotic cells were detected by a CytoFlex flow cytometer (Beckman Coulter, Inc., Brea, CA, USA) within 1 h.

### 4.7. Immunofluorescence Analysis

After deparaffinization of jejunal tissue sections fixed with 4% paraformaldehyde, ethylenediaminetetraacetic acid (EDTA, 1 mol/L, pH 9.0, Gooddbio Technology CO., LTD., Wuhan, China) was added to extract antigens. Tissues were permeabilized with 0.5% Triton X-100, and blocked with 3% bovine serum albumin (BSA), before being incubated with the ZO-1 rabbit polyclonal antibody (1:200; Abcam Plc., Cambridge, UK), goat anti-rabbit IgG-FITC (Gooddbio Technology Co., Ltd., Wuhan, China). Next, the nucleus was stained with 4’-6-diamidino-2-phenylindole (DAPI, Gooddbio Technology Co., Ltd., Wuhan, China) in the dark, and the excess DAPI were washed away for confocal laser scanning. Microscopic observation was followed by statistical analysis image-pro plus 6.0 (Media Cybernetics, Inc., Rockville, MD, USA).

### 4.8. Immunohistochemistry Analysis of Mucosal sIgA

After dewaxing the samples, the sections were placed in xylene and various grades of absolute ethanol in sequence. After washing with distilled water, antigen retrieval was performed with citric acid retrieval solution for 10 min, and the sections were naturally cooled and washed with PBS, before being sequentially incubated with 3% H_2_O_2_ and 10% goat serum in the dark. After discarding the blocking solution, the sections were incubated with antibody sIgA. DAB color was developed, and staining was terminated with distilled water, followed by hematoxylin counterstaining, 1% hydrochloric acid alcohol differentiation and ammonia back to blue. Finally, the sections were dehydrated and dried with graded alcohol, and then sealed with neutral gum once the xylene was transparent.

### 4.9. RNA Isolation, Reverse Transcription, and Real-Time Quantitative PCR

Total RNA of mucosa was extracted using Trizol and was reverse transcribed into cDNA with a PrimeScript™ RT reagent kit with gDNA Eraser (Takara Biotechnology Co., Ltd., Dalian, China). This process was performed as follows: I: 37 °C for 15 min; II: 85 °C for 5 s. qPCR was performed with the SYBR^®^ Green PCR I PCR reagents (Takara). The procedure of q-PCR was performed as follows: 95 °C for 30 s, followed by 40 cycles at 95 °C for 5 s and 60 °C for 30 s. The RNA expression levels of target genes were analyzed using the 2^−ΔΔCt^ method with β-actin as internal reference [65]. All primers used are showed in Table S2.

### 4.10. Fecal Bacterial Quantification

Total DNA was extracted from cecum digesta with the Stool DNA Kits (Omega Bio-Tek, Doraville, CA, USA), executed by real-time quantitative PCR using the CFX96 Real-Time PCR Detection system (Bio-Rad Laboratories, Hercules, CA, USA). The total bacteria application program entailed 95 °C for 25 s, followed by 40 cycles of 95 °C for 5 s and 64.5 °C for 25 s. *Lactobacillus*, *E. coli*, *Bacillus* and *Bifidobacterium* were tested using the SuperReal PreMix (Probe) kit (Tiangen Biotech Co., Ltd., Beijing, China). All results are included for 95 °C for 15 min, followed by 50 cycles of 95 °C for 3 s, and 53 °C for 25 s. All primers used are showed in Table S3.

### 4.11. Statistical Analysis

The data were analyzed using two-way ANOVA with the General Linear Model (GLM) procedure of SPSS as a 2 (BGL) × 2 (ETEC) factorial design. *p*-value < 0.05 was deemed to be significant, and 0.05 < *p*-value < 0.1 was deemed to show a significant trend. Duncan’s multiple range test was used based on ANOVA to show a significant difference. All data were analyzed using SPSS 27.0 (IBM, Chicago, IL, USA). All results are expressed as means with standard errors.

## 5. Conclusions

In conclusion, our results indicated a positive effect of dietary BGL supplementation on the intestinal health in the weaned pigs upon ETEC challenge. This may be connected with suppression of inflammation and improved intestinal immunity and antioxidant capacity, as well as improved intestinal microbiota.

## Figures and Tables

**Table 1:** Effect of BGL supplementation on ADFI and ADG in weaned pigs during the experiment.

	Treatments				SEM	*p*-Value		
	CON	BGL	ECON	EBGL		BGL	ETEC	Interaction
ADFI (g/d)	449.03	447.71	467.96	474.49	22.15	0.96	0.64	0.94
ADG (g/d)	306.12	289.64	303.43	324.52	14.35	0.94	0.60	0.55
F:G	1.50	1.55	1.53	1.47	0.04	0.96	0.78	0.47
Diarrhea rate (%)	28.57	28.57	38.09	33.33	7.01	0.68	0.41	0.68

ADFI, average daily feed intake; ADG, average daily gain; F:G, feed: gain ratio. Mean and total SEM are listed in separate columns (*n* = 8). CON, pigs were fed with a basal diet; BGL, pigs were fed with a BGL-containing diet (500 mg/kg); ECON, pigs were fed with a basal diet and challenged by ETEC; EBGL, pigs were fed with a BGL-containing diet and challenged by ETEC. These notes also apply to the following tables.

**Table 2:** Effect of BGL on intestinal antioxidant in weaned pigs upon ETEC challenge.

	ITEM	Treatments				SEM	*p*-Value		
		CON	BGL	ECON	EBGL		BGL	ETEC	Interaction
Duodenum	MDA (nmol/mL)	1.03 ^ab^	0.69 ^b^	1.51 ^a^	0.79 ^b^	0.12	0.03	0.20	0.39
	T-AOC (U/mL)	0.48	0.42	0.4	0.49	0.03	0.79	0.92	0.21
	GSH-PX (U)	175.95 ^a^	184.42 ^a^	139.72 ^b^	163.59 ^ab^	6.60	0.19	0.03	0.53
	CAT (U/mL)	81.95 ^ab^	75.01 ^b^	62.86 ^b^	103.76 ^a^	4.84	0.55	0.046	<0.01
	T-SOD (U/mL)	123.57 ^ab^	110.63 ^b^	136.51 ^a^	115.02 ^ab^	3.34	<0.01	0.14	0.46
Jejunum	MDA (nmol/mL)	1.03 ^ab^	0.8 ^ab^	1.36 ^a^	0.67 ^b^	0.1	0.04	0.77	0.16
	T-AOC (U/mL)	0.57 ^a^	0.43 ^ab^	0.30 ^b^	0.44 ^ab^	0.03	0.99	0.048	0.04
	GSH-PX	303.82	312.86	295.83	304.95	18.79	0.82	0.85	0.99
	CAT (U/mL)	52.1	57.3	47.63	53.83	3.61	0.46	0.61	0.95
	T-SOD (U/mL)	409.22 ^a^	347.16 ^ab^	297.27 ^b^	307.46 ^b^	13.58	0.25	<0.01	0.12
Ileum	MDA (nmol/mL)	1.82 ^b^	1.77 ^b^	2.64 ^a^	1.30 ^b^	0.15	<0.01	0.45	0.01
	T-AOC (U/mL)	0.38 ^b^	0.59 ^a^	0.27 ^b^	0.35 ^b^	0.03	0.01	<0.01	0.24
	GSH-PX	201.69 ^ab^	215.34 ^ab^	175.60 ^b^	223.13 ^a^	7.61	0.04	0.53	0.25
	CAT (U/mL)	44.35 ^ab^	50.23 ^a^	28.79 ^c^	32.11 ^bc^	2.73	0.31	<0.01	0.77
	T-SOD (U/mL)	122.06 ^c^	165.63 ^a^	116.13 ^c^	148.87 ^b^	4.76	<0.01	0.01	0.21

MDA, Malondialdehyde; T-AOC, total antioxidant capacity; GSH-Px, Glutathione peroxidase; CAT, catalase; T-SOD, total superoxide dismutase. Values within a row with different letters differ significantly (*p* < 0.05).

**Table 3:** Effect of BGL supplementation on intestinal microbiota and metabolites in weaned pigs upon ETEC challenge.

ITEM	Treatments				SEM	*p*-Value		
	CON	BGL	ECON	EBGL		BGL	ETEC	Interaction
microbial populations (lg(copies/g))								
Total bacteria	11.51	11.57	11.47	11.58	0.03	0.19	0.78	0.71
*Escherichia coli*	8.51 ^b^	8.52 ^b^	9.49 ^a^	9.84 ^a^	0.18	0.54	0.01	0.55
*Lactobacillus*	8.21 ^bc^	8.83 ^ab^	7.94 ^c^	8.99 ^a^	0.15	0.00	0.82	0.40
*Bifidobacterium*	6.18	6.56	6.55	6.17	0.11	0.99	0.98	0.11
*Bacillus*	9.10 ^ab^	9.45 ^a^	8.95 ^b^	9.38 ^a^	0.07	0.01	0.40	0.76
VFA (g/g)								
Acetic acid	2.20	2.18	2.03	2.56	0.11	0.26	0.64	0.23
Propanoic acid	1.13 ^ab^	1.14 ^ab^	0.79 ^b^	1.43 ^a^	0.09	0.05	0.88	0.06
Butyric acid	0.64	0.70	0.58	0.67	0.05	0.43	0.67	0.85

Values within a row with different letters differ significantly (*p* < 0.05). VFA, volatile fatty acid.

## Data Availability

Not applicable.

## Supplementary Materials

The following supporting information can be downloaded at: https://www.mdpi.com/article/10.3390/ijms231810290/s1.
