# Supplementary material for: Agrobacterium sp. ZX09 β-Glucan Attenuates Enterotoxigenic Escherichia coli-Induced Disruption of Intestinal Epithelium in Weaned Pigs
Source: Int J Mol Sci. 2022 Sep 7;23(18):10290. doi: 10.3390/ijms231810290 (PMC9499454; doi:10.3390/ijms231810290)
Supplement: Supplementary file 1 [file ijms-23-10290-s001.zip › Table S1.pdf]

Table S1. Experiment basal diet composition and nutrient level.

Ingredients	%	Nutrient level	contents
Corn	28.31	Digestible energy (calculated, MJ/kg)	14.78
Extruded corn	24.87	Crude Protein (%)	19.68
Soybean meal	8.5	Calcium (%)	0.81
Extruded full-fat soybean	10.3	Available phosphorus (%)	0.55
Fish meal	4.2	Lysine	1.35
Whey powder	7	Methionine	0.42
Soybean protein concentrate	8	Methionine + cysteine	0.6
Soybean oil	2	Threonine	0.79
Sucrose	4	Tryptophan	0.22
Limestone	0.9		
Dicalcium phosphate	0.5		
NaCl	0.3		
L -Lysine HCl (78%)	0.47		
DL-Methionine	0.15		
L -Threonine (98.5%)	0.13		
Tryptophan (98%)	0.03		
Chloride choline	0.1		
Vitamin premix [1]	0.04		
Mineral premix [2]	0.2		
Total	100		

[1]The vitamin premix provided the following per kg of diet: 9000 IU of VA, 3000 IU of VD 3, 20 IU of VE, 3 mg of VK 3, 1.5 mg of VB1, 4 mg of VB 2, 3 mg of VB6, 0.02 mg of VB12, 30 mg of niacin, 15 mg of pantothenic acid, 0.75 mg of folic acid, and 0.1 mg of biotin. [2] The mineral premix provided the following per kg of diet: 100 mg Fe, 6 mg Cu, 100 mg Zn, 4 mg Mn, 0.30 mg I, 0.3 mg Se.

[3]The diet was formulated based on the recommendation of NRC2012.
