# Supplementary material for: Agrobacterium sp. ZX09 β-Glucan Attenuates Enterotoxigenic Escherichia coli-Induced Disruption of Intestinal Epithelium in Weaned Pigs
Source: Int J Mol Sci. 2022 Sep 7;23(18):10290. doi: 10.3390/ijms231810290 (PMC9499454; doi:10.3390/ijms231810290)
Supplement: Supplementary file 1 [file ijms-23-10290-s001.zip › Table S2.pdf]

Table S2. Primers sequences used for quantitative RT-PCR

Gene	Primer sequence (5’ –3’)	Product size (bp)
β-Actin	F: TGGAACGGTGAAGGTGACAGC<br>R: GCTTTTGGGAAGGCAGGGACT	177
Caspase3	F: GGGATTGAGACGGACAGTGG<br>R: TGAACCAGGATCCGTCCTTTG	136
Caspase9	F: AATGCCGATTTGGCTTACGT<br>R: CATTTGCTTGGCAGTCAGGTT	195
Bax	F: AAGCGCATTGGAGATGAACT<br>R: TGCCGTCAGCAAACATTTC	121
Bcl-2	F: ATGTGTGTGGAGAGCGTCAA<br>R: GCCCATACAGCTCCACAAAG	142
Keap1	F: ACGACGTGGAGACAGAAACGT<br>R: GCTTCGCCGATGCTTCA	56
NR-F2	F: GCCCCTGGAAGCGTTAAAC<br>R: GGACTGTATCCCCAGAAGGTTGT	67
Ho-1	F: CGCTCCCGAATGAACAC<br>R: GCTCCTGCACCTCCTC	112
TNF-α	F: GCATCGCCGTCTCCTACCAG<br>R: GGGCAGGTTGATCTCGGCAC	173
IL-6	F: TGGCTACTGCCTTCCCTACC<br>R: CACACATCTCCTTTCTCATTGC	153
IL-10	F: TAATGCCGAAGGCAGAGAGT<br>R: CTGCATCCACTTCCCAACCA	77
TLR4	F: AGAAACTCTTCACTGGGCCG<br>R: TCCAGGTTGGGCAGGTTAGA	152
MyD88	F: CCATTCGAGATGACCCCCTG<br>R: TAGCAATGGACCAGACGCAG	183
NF-kB	F: GTGTGTAAAGAAGCGGGACCT<br>R: CACTGTCACCTGGAAGCAGAG	139
