# Supplementary material for: Agrobacterium sp. ZX09 β-Glucan Attenuates Enterotoxigenic Escherichia coli-Induced Disruption of Intestinal Epithelium in Weaned Pigs
Source: Int J Mol Sci. 2022 Sep 7;23(18):10290. doi: 10.3390/ijms231810290 (PMC9499454; doi:10.3390/ijms231810290)
Supplement: Supplementary file 1 [file ijms-23-10290-s001.zip › Table S3.pdf]

Table S3. Sequences of primers and probes for quantitative real-time polymerase chain reaction

Item	Primer and probe sequences（5′-3′）	Annealing temperature (℃)	Size (bp)
Total bacteria	F: ACTCCTACGGGAGGCAGCAG<br>R: ATTACCGCGGCTGCTGG	60	200
Lactobacillus	F: GAGGCAGCAGTAGGGAATCTTC<br>R: CAACAGTTACTCTGACACCCGTTCTTC<br>P:AAGAAGGGTTTCGGCTCGTAAAACTCTGTT	60	126
Escherichia coli	F: CATGCCGCGTGTATGAAGAA<br>R: CGGGTAACGTCAATGAGCAAA<br>P: AGGTATTAACTTTACTCCCTTCCTC	60	96
Bifidobacterium	F: CGCGTCCGGTGTGAAAG<br>R: CTTCCCGATATCTACACATTCCA<br>P: ATTCCACCGTTACACCGGGAA	60	121
Bacillus	F: GCAACGAGCGCAACCCTTGA<br>R: TCATCCCCACCTTCCTCCGGT<br>P: CGGTTTGTCACCGGCAGTCACCT	60	92
